# Improving Image-Based Plant Disease Classification With Generative Adversarial Network Under Limited Training Set

**DOI:** 10.3389/fpls.2020.583438

**Published:** 2020-12-04

**Authors:** Luning Bi, Guiping Hu

**Affiliations:** Department of Industrial and Manufacturing Systems Engineering, Iowa State University, Ames, IA, United States

**Keywords:** plant disease, classification, regularization, convolutional neural network, generative adversarial network

## Abstract

Traditionally, plant disease recognition has mainly been done visually by human. It is often biased, time-consuming, and laborious. Machine learning methods based on plant leave images have been proposed to improve the disease recognition process. Convolutional neural networks (CNNs) have been adopted and proven to be very effective. Despite the good classification accuracy achieved by CNNs, the issue of limited training data remains. In most cases, the training dataset is often small due to significant effort in data collection and annotation. In this case, CNN methods tend to have the overfitting problem. In this paper, Wasserstein generative adversarial network with gradient penalty (WGAN-GP) is combined with label smoothing regularization (LSR) to improve the prediction accuracy and address the overfitting problem under limited training data. Experiments show that the proposed WGAN-GP enhanced classification method can improve the overall classification accuracy of plant diseases by 24.4% as compared to 20.2% using classic data augmentation and 22% using synthetic samples without LSR.

## Introduction

With the increasing global population, the demand for agriculture production is rising. Plant diseases cause substantial management issues and economic losses in the agricultural industry (Abu-Naser et al., 2010). It has been reported that at least 10% of global food production is lost due to plant disease (Strange and Scott, 2005). The situation is becoming increasingly complicated because climate change alters the rates of pathogen development and diseases are transferred from one region to another more easily due to the global transportation network expansion (Sladojevic et al., 2016). Therefore, early detection, timely mitigation, and disease management are essential for agriculture production (Barbedo, 2018a).

Traditionally, plant disease inspection and classification have been carried out through optical observation of the symptoms on plant leaves by human with some training or experience. Plant disease recognition has known to be time-consuming and error-prone. Due to the large number of cultivated plants and their complex physiological symptoms, even experts with rich experience often fail to diagnose specific diseases and consequently lead to mistaken disease treatments and management (Ferentinos, 2018).

Many methods have been developed to assist disease recognition and management. Lab-based techniques have been developed and established in the past decades. The commonly used techniques for plant disease recognition include enzyme-linked immunosorbent assay (ELISA), polymerase chain reaction (PCR), immunoflourescence (IF), flow cytometry, fluorescence in situ hybridization (FISH), and DNA microarrays (Sankaran et al., 2010). However, these techniques require an elaborate procedure and consumable reagents. Meantime, image-based machine learning methods for plant disease recognition, which identify plant diseases by training computers with labeled plant images, have become popular. The advantages of image recognition include: (1) the ability to deal with a large number of input parameters, i.e., image pixels, (2) the minimization of human errors, and (3) the simplified process (Patil and Kumar, 2011).

The key to improving the plant disease recognition accuracy is to extract the right features of the surface of plant leaves (Naresh and Nagendraswamy, 2016; Zhang and Wang, 2016). The emergence of deep learning techniques has led to improved performance. Although deep learning based models take a long time to train, its testing time is fast because all information from the training dataset has been integrated into the neural network (Kamilaris and Prenafeta-Boldú, 2018). For the agricultural applications, convolutional neural networks (CNN) have been used for image recognition (Lu et al., 2017). Dhakate et al. used a convolutional neural network for the recognition of pomegranate plant diseases and achieved 90% overall accuracy (Dhakate and Ingole, 2015). Ghazi et al. proposed a hybrid method of GoogLeNet, AlexNet, and VGGNet to classify 91,758 labeled images of different plant organs. Their combined system achieved an overall accuracy of 80% (Ghazi et al., 2017). Ferentinos developed CNN models to classify the healthy and diseased plants using 87,848 images. The success rate was significantly high which can reach 99.53% (Ferentinos, 2018). Ma et al. proposed a deep CNN to recognize four cucumber diseases. The model was trained using 14,208 images and achieved an accuracy of 93.4% (Ma et al., 2018). With the high classification accuracy, it can be concluded that CNNs on leave images are highly suitable for plant disease recognition (Grinblat et al., 2016).

It should be noted that the high prediction accuracy is predicated on that thousands of labeled images were used to train CNNs. A major problem often facing the automatic identification of plant diseases with CNNs is the lack of labeled images capable of representing the wide variety of conditions and symptom characteristics found in practice (Barbedo, 2019). Experimental results indicate that while the technical constraints linked to automatic plant disease classification have been largely overcome, the use of limited image datasets for training brings many undesirable consequences that still prevent the effective dissemination of this type of technology (Barbedo, 2018b). Real datasets often do not have enough samples for deep neural networks to properly learn the classes and the annotation errors, which may damage the learning process (Barbedo, 2018a). If the model learns to assign a full probability to the ground truth label for each training example, it is not guaranteed to generalize because the model becomes too confident about its predictions (Szegedy et al., 2016). It should be noted that although it is relatively cheap to collect images, using additional unlabeled data is non-trivial to avoid model overfitting. This serves as the major motivation for this study on developing a new method that can address the plant disease classification with limited labeled training images.

Data augmentation using synthetic images is the most common method used in training CNN with small amounts of data (Emeršic et al., 2017). Hu et al. synthesized face images by compositing the automatically detected face parts from two existing subjects in the training set. Their method improved over the state-of-the-art method with a 7% margin (Hu et al., 2017). Guo et al. merged the training set with another dataset from the same domain and obtained a performance improvement of 2% (Guo and Gould, 2015). Papon et al. proposed a rendering pipeline that generates realistic cluttered room scenes for the classification of furniture classes. Compared to using standard CNN, the proposed method improved the classification accuracy by up to 2% (Papon and Schoeler, 2015). These methods generate synthetic images by extracting and recombining of local regions of different real images.

In this study, we designed a generative adversarial network (GAN) to generate completely new synthetic images to enhance the training set. GAN was designed based on game theory to generate additional samples with the same statistics as the training set. Compared with the methods in the existing literature, GAN is capable to generate full synthetic images that can increase the diversity of the dataset. Therefore, it has become an increasingly popular tool to address the limited dataset issue (Goodfellow et al., 2014). Nazki et al. (2020) proposed Activation Reconstruction (AR) – GAN to generate synthetic samples of high perceptual quality to reduce the partiality introduced by class imbalance. Compared with Nazki’s work which considered 9 classes of images with about 300 images in each category, our work has considered a more stringent situation of limited dataset which includes 38 classes with 10-28 images in each category. Therefore, one of the key objectives of this study is to reduce overfitting of the model. Label smoothing regularization (LSR) is introduced in this paper. In addition to maximizing the predicted probability of the truth-ground class, LSR also maximizes the predicted probability of the non-truth ground classes (Szegedy et al., 2016). Similarly, Xie et al. (2016) proposed a method named DisturbLabel which prevents the overfitting problem by adding label noises to the CNN. Pereyra et al. (2017) found out that label smoothing can improve the performance of the models on benchmarks without changing other parameters. In our paper, Wasserstein generative adversarial network with gradient penalty (WGAN-GP) is combined with LSR to generate images that can enlarge the training dataset and regularize the CNN model simultaneously.

The main contributions of this study lie in two dimensions:

1. To improve the generalization of the proposed method, multiple diseases and multiple plant types have been considered in this paper. The majority of the existing studies focused on a single type of disease or only one plant type. In reality, there may exist multiple diseases for one plant type. However, in reality, it is often necessary to detect the multiple diseases of multiple plant types. Therefore, it would be preferable to design recognition methods with the capability to address the multi-disease and multi-plant type situation.
2. To address the issue of limited training set, an approach that combines classical data augmentation and synthetic augmentation is proposed. LSR has also been employed to increase the generalization ability of the model. Four experiments have been conducted to validate the effectiveness of each component in the proposed framework. The results show that compared to the classic data augmentation methods, the proposed method can improve the total accuracy by 4.2%.

The rest of this paper is organized as follows. Section 2 introduces the motivation of this paper and the structure of the proposed regularized GAN-based approach. Section 3 includes a case study, the experiment results and comparisons. Finally, the paper concludes with the summary, findings, and future research directions in Section 4.

## Materials and Methods

Image-based plant disease recognition techniques have been developed with the reduced cost for image collection and the increased computational resources. However, in many situations for plant disease, there is not enough well-labeled data due to the high cost of data annotation. Under these circumstances, the machine learning models are prone to overfitting and fail to make accurate classifications for new observations. This study aims to achieve high plant disease classification accuracy with limited training dataset.

### Framework of the Proposed Method

**FIGURE 1:**
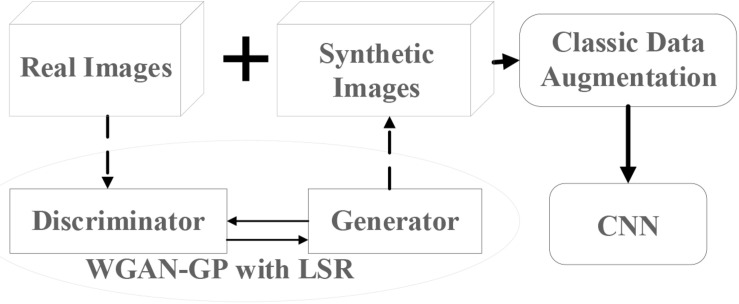
Framework of the proposed method.

To improve the prediction accuracy of CNN in the classification of plant diseases using a limited training dataset, three techniques have been designed and implemented in this study, i.e., data augmentation, WGAN-GP, and LSR. The framework of the proposed method is shown in Figure 1. The first step is to train the WGAN-GP with LSR using real images. The trained WGAN-GP is then used to generate additional labeled images. The synthetic images will be mixed with real images and then augmented through classic data augmentation methods. Finally, the combined dataset will be used to train the CNN. In the following few sections, we will discuss each of the components in detail.

### Convolutional Neural Networks (CNN)

Convolutional Neural Networks is used as the supporting framework of our method. CNN is a class of deep, feed-forward artificial neural networks. It was adopted widely for its fast deployment and high performance on image classification tasks. CNNs are usually composed of convolutional layers, pooling layers, batch normalization layers and fully connected layers. The convolutional layers extract features from the input images whose dimensionality is then reduced by the pooling layers. Batch normalization is a technique used to normalize the previous layer by subtracting the batch mean and dividing by the batch standard deviation, which can increase the stability and improve the computation speed of the neural networks. The fully connected layers are placed near the output of the model. They act as classifiers to learn the non-linear combination of the high-level features and to make numerical predictions. Detailed descriptions on each type of function can be accessed from Gu et al. (2018).

It should be noted that CNN requires a large training dataset, which is typically not the case for plant disease recognitions. With the number of model parameters is greater than the number of data samples, a small training dataset will lead to the overfitting problem, which results from a model that responds too closely to a training dataset and fails to fit additional data or predict future observations reliably. One of the commonly adopted methods to address this problem is data augmentation.

### Data Augmentation

**FIGURE 2:**
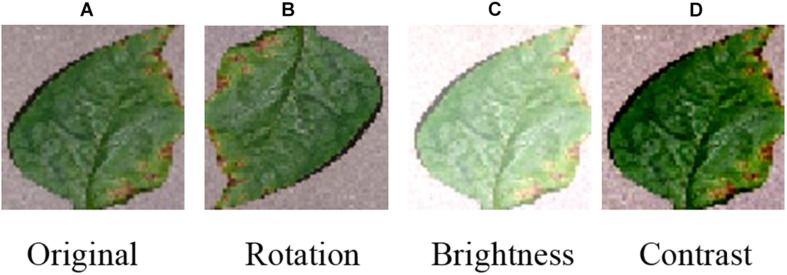
Classic data augmentation methods. **(A)** Original, **(B)** Rotation, **(C)** Brightness, and **(D)** Contrast.

Data augmentation is a method to increase the number of labeled images. The classic data augmentation methods include vertical flipping, horizontal flipping, 90° counterclockwise rotation, 180° rotation, 90° clockwise rotation, random brightness decrease, random brightness increase, contrast enhancement, contrast reduction and sharpness enhancement. Figure 2 lists the examples of original image (Figure 2A), rotation (Figure 2B), brightness increase (Figure 2C), and contrast increase (Figure 2D).

Although data augmentation techniques decrease the impact of the limited training dataset problem, they cannot reproduce most of the practical diversity. This is also the reason why the generative adversarial network has been incorporated in this study.

### Wasserstein Generative Adversarial Network (WGAN)

Unlike regular data augmentation methods, GAN is able to generate new images for training, which increases the diversity of data. GANs were firstly introduced by Ian Goodfellow et al. (2014). The generative adversarial networks (GANs) consist of two sub-networks: a generator and a discriminator. The generator captures the training data distribution while the discriminator estimates the probability that an image came from the training data rather than the generator.

(1) $$\begin{array}{c} \min_{G}\max_{D}\,V\,\left(D,G\right)=E_{x-p_{d\,a\,t\,a}\,\left(x\right)}\,\left[\log D\,\left(x\right)\right]\\ +E_{z-p_{\text{Noise}\,\left(z\right)}}[\log\left(1-D\left(G\left(z\right)\right)\right] \end{array}$$

Where *D* represents the discriminator network, *G* is the generator network, *z* is a noise vector drawn from a distribution *p*_*Noise(z)*_, *x* is a real image drawn from the original dataset *p*_*data(x)*_.

**FIGURE 3:**
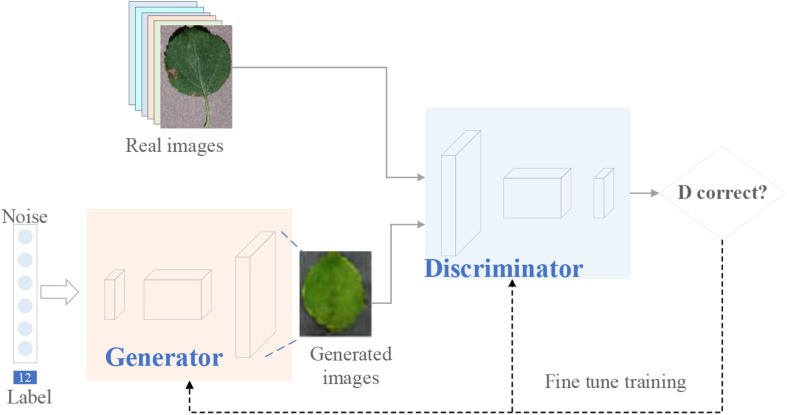
Training process of the original GAN.

The idea behind Eq. (1) is that it increases the ability of the generator to fool the discriminator which is trained to distinguish synthetic images from real images. The training process of the original GAN is shown in Figure 3. The specific steps are as follows.

1. Initialize the parameters of the generator and the discriminator.
2. Sample a batch of noise samples for the generator. Usually, uniform distribution or Gaussian distribution is used.
3. Use the generator to transform the noise samples and predefined labels into images that are labeled as fake.
4. The real images are labeled as true. Then the real images and the synthetic images are mixed and used as the input of the discriminator.
5. Train the discriminator to improve the ability to classify the synthetic images and the real images.
6. Train the generator to generate more images that will be discriminated as true by the generator.
7. Repeat step 2 - step 6 until the termination condition is satisfied.

Many variants of GAN have been proposed in the past several years. Mirza et al. proposed the conditional GAN, which can provide better representations for multimodal data generation (Mirza and Osindero, 2014). Radford et al. proposed the deep convolutional GAN (DCGAN), which allows training a pair of deep convolutional generator and discriminator networks (Radford et al., 2015). Arjovsky et al. (2017) proposed the Wasserstein GAN (WGAN) which uses Wasserstein distance to provide gradients that are useful for updating the generator. Even though the WGAN performs more stable in the training process, it sometimes fails to converge due to the use of weight clipping. Therefore, Gulrajani et al. (2017) proposed an improved version of WGAN in which the weight clipping is replaced by the gradient penalty.

**FIGURE 4:**
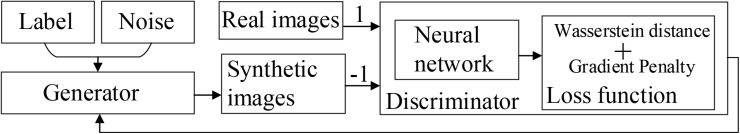
Training process of the WGAN-GP. The real images are labeled as “1”. The synthetic images are labeled as “-1”. The Wasserstein distance and gradient penalty are used in the loss function.

As shown in Figure 4, the major differences between the implementation of WGAN-GP and the original GAN include two aspects. The first is that the WGAN-GP uses the Wasserstein loss function with gradient penalty. Compared with the Jensen–Shannon (JS) and Kullback–Leibler (KL) divergence used in the DCGAN, Wasserstein distance can measure the distance between the distribution of real images and fake images, which can help improve the convergence of the network. The second is that in the WGAN-GP, the real and fake images are labeled as 1 and -1, while in the DCGAN, they are labeled as 1 and 0. This encourages the discriminator (critic) to output scores that are different for real and fake images.

### WGAN–GP With Label Smoothing Regularization (WGAN-GP-LSR)

In this paper, we made two changes to the WGAN-GP. The first is that we combined the conditional GAN and the WGAN-GP so that the generator can generate images of specific labels. For the generator, the input is a noise vector and a predefined label. Firstly, the label will be represented following the one-hot encoding method. Then the label will be converted to a vector that has the same size as the noise vector by multiplying a matrix. In practice, we used the built-in embedding function of Keras in which each input integer label is used as the index to access a table that contains all possible vectors. The final input vector is obtained by conducting an element wise multiply operation between the noise vector and the label vector. The generator is basically a neural network that outputs matrices of the image size with one matrix representing one image. For the discriminator, the output includes the class labels and the validity labels. The second is that LSR is used to modify the loss function of GAN. Compared with L1 and L2 regularization methods which change the weights, LSR directly influences the output of the network through the loss function. At the same time, LSR can increase the robustness of GAN and help avoid model collapse.

In the training of GAN, the most widely used loss function for multiclass classification tasks is the cross-entropy loss as Eq. (2),

(2) $$L=-\sum_{i=1}^{N}\log\left(p\,\left(i\right)\right)\,q\,\left(i\right)$$

where *i* is the index of the disease type, *N* is the total number of disease types, *p*(*i*) is the predicted probability of the image belonging to class *i*, *q*(*i*) equals to 1 if the label of the image is *i*; otherwise, *q*(*i*) equals to 0.

The minimization of the cross-entropy loss is achieved when the predicted probability of ground-truth classes is maximum. However, if the model assigns full probabilities to ground-truth labels, it is likely to be overfitted. In other words, it will be very easy for CNN to determine the truth-ground classes of the images. It means that the improvement brought by generating additional images for training will be limited. Thus, the regularization is introduced. Regularization is a technique that makes the model less confident such that the model generalizes better.

The LSR method is used in this paper. The objective function of GAN is as Eq. (3) (Szegedy et al., 2016),

(3) $$L_{L\,S\,R}=-\left(1-\varepsilon \right)\,\log\left(p\,\left(y\right)\right)-\frac{\varepsilon }{N}\,\sum_{i=1}^{N}\log\left(p\,\left(i\right)\right)$$

where *ε* is a hyperparameter between 0 and 1, *i* is the index of the disease type, *N* is the total number of disease types, *p*(*i*) is the predicted probability of the image belonging to non-truth ground class *i*, *p(y)* is the predicted probability of the image belonging to truth-ground class *y*.

If εis equal to 0, Eq. (3) is the same as Eq. (2) since the second term in Eq. (3) becomes 0. The objective is to maximize the predicted probability of the truth-ground class. If εis equal to 1, the first term equals to 0. The objective is to maximize the summation of the predicted probability of the other non-truth ground classes. Therefore, in addition to maximizing the predicted probability of the truth-ground class, the LSR function also maximizes the predicted probability of the other non-truth ground classes. In the training process of the generator, the synthetic images will learn the same distribution of the probability. In other words, each generated image contains the features of all disease types, which can improve the generalization ability of the model. In practice, a generated image will be assigned with the label of the largest predicted possibility.

## Case Study

To validate the effectiveness of the proposed method, a case study on plant disease classification has been conducted. The dataset contains images of different plant diseases from multiple species. Four experiments were conducted to compare the results. In Experiment I, the CNN was trained without data augmentation. In Experiment II, the CNN was trained with classic data augmentation methods. In Experiment III, the CNN was trained with classic augmentation methods and WGAN-GP. In Experiment IV, the CNN was trained with classic data augmentation methods and WGAN-GP-LSR.

### Data Source and Performance Measure

The dataset used in this paper is from www.plantvillage.org. The original dataset contains 43,843 labeled images. To imitate the limited dataset problem, we randomly selected 873 images (i.e., 1.9% of all available images) as the training dataset. For each category, there are 10-28 images for training. We also randomly selected 4,384 images (i.e., 10% of all available images) as the testing dataset. This step was completed by using the *train_test_split* function from sklearn package. As shown in Table 1, the images include 14 crop species: Apple, Blueberry, Cherry, Corn, Grape, Orange, Peach, Bell Pepper, Potato, Raspberry, Soybean, Squash, Strawberry, and Tomato. It contains images of 17 fungal diseases, 4 bacterial diseases, 2 mold (Oomycete) diseases, 2 viral diseases, and 1 disease caused by a mite. Twelve crop species also have images of healthy leaves that are not visibly affected by a disease (Hughes and Salathé, 2015). The total number of classes is 38 which includes 12 groups of healthy leaves and 26 groups of diseased leaves.

**TABLE 1:** Dataset for classification of plant disease.

**Specie**	**Class**	***N*_1_**	***N*_2_**	**Specie**	**Class**	***N*_1_**	***N*_2_**
	1. Botryospaeria obtuse	10	46	Potato	14. Alternaria solani	16	81
Apple	2. Venturia inaequalis	13	58		15. Phytophthora Infestans	24	58
	3. Gymnosporangium juniperi-virginianae	15	30		H. Healthy	22	15
	A. Healthy	20	162	Squash	16. Erysiphe cichoracearum	28	168
Blueberry	B. Healthy	22	117	Strawberry	17. *Diplocarpon earlianum*	25	65
Cherry	4. *Podosphaera* spp.	19	98		I. Healthy	28	40
	C. Healthy	14	76	Raspberry	J. Healthy	19	51
	5. *Cercospora zeae-maydis*	27	32	Soybean	K. Healthy	28	378
Corn	6. *Puccinia sorghi*	25	90	Tomato	18. *Xanthomonas campestris* pv. vesicatoria	27	163
	7. *Exserohilum turcicum*	24	69		19. Alternaria solani	25	93
	D. Healthy	27	85		20. Phytophthora Infestans	28	142
	8. *Guignardia bidwellii*	28	94		21. Fulvia fulva	24	70
Grape	9. *Phaeomoniella* spp.	21	117		22. *Septoria lycopersici*	28	136
	10. *Pseudocercospora vitis*	26	90		23. *Tetranychus urticae*	27	149
	E. Healthy	26	31		24. *Corynespora cassiicola*	21	121
Orange	11. *Candidatus Liberibacter*	28	467		25. Mosaic Virus	20	416
Peach	12. *Xanthomonas campestris*	27	187		26. Yellow leaf curl virus	25	26
	F. Healthy	24	26		L. Healthy	24	136
Pepper	13. *Xanthomonas campestris*	16	96				
	G. Healthy	22	105				

*N_1_ represents the number of training images. *N*_2_ represents the number of testing images. The healthy classes are numbered from A to L. The diseased classes are numbered from 1 to 26.*

Four measurements have been used as the performance indicators in this study, i.e., overall accuracy, precision, recall, and F_1_ score. The overall accuracy, recall and precision can be calculated as in Eq. (4)–Eq. (6).

(4) $$\mathrm{Overall}\,\mathrm{Accuracy}=\frac{\mathrm{True}\,\mathrm{Positive}+\mathrm{True}\,\mathrm{Negative}}{\text{Total}}$$

(5) $$\mathrm{Recall}=\frac{\mathrm{True}\,\mathrm{Positive}}{\mathrm{True}\,\mathrm{Positive}+\mathrm{False}\,\mathrm{Negative}}$$

(6) $$\mathrm{Precision}=\frac{\mathrm{True}\,\mathrm{Positive}}{\mathrm{True}\,\mathrm{Positive}+\mathrm{False}\,\mathrm{Positive}}$$

Since the problem is a multi-class classification problem, a modification on recall and precision calculations has been made as Eq. (7) and Eq. (8). The F_1_ score is the harmonic mean of the recall and precision which can be calculated based on Eq. (9).

(7) $${\text{Recall}}_{i}=\frac{M_{i\,i}}{\sum_{j}M_{i\,j}}$$

(8) $${\text{Precision}}_{i}=\frac{M_{i\,i}}{\sum_{j}M_{j\,i}}$$

(9) $${\text{F}}_{1}\,{\mathrm{score}}_{i}=\frac{2\times {\text{Recall}}_{i}\times {\text{Precision}}_{i}}{{\text{Recall}}_{i}+{\text{Precision}}_{i}}$$

Where *M*_*ij*_ is the number of images belonging to the *i*th category that are predicted to be in the *j*th category, ∑_*j*_*M*_*ij*_ is the number of samples belonging to the *i*th category, Recall*_*i*_* is the ratio of samples belonging to the *i*th category that are correctly classified, Presion*_*i*_* is the ratio of samples predicted to be in the *i*th category that are correctly classified.

### Parameters of Neural Networks

The architectures of the generator and the discriminator are shown in Table 2. For the generator, we established a network with a 1000-dimensional vector input. The inputs consist of two parts, i.e., noise and label. The noise is a vector of 1000 randomly generated variables. The label is converted to a vector of size using the built-in embedding function in Keras. In the function, each integer label is used as the index to access a table that contains all possible vectors. Then the input can be obtained by conducting element-wise multiplication on the two 1000-dimensional vectors. A dense layer is then used to covert the input vector to a vector of size 128 × 16 × 16. Through three convolutional layers, the output is an image of dimension 128 × 128 × 3. For the discriminator, all input images have been resized to 128 × 128 × 3. The real images are assigned with label “1” while the synthetic images are assigned with label “-1”. There are two output layers. One output layer has one neuron telling whether the input image is real or fake. The other output layer has 38 neurons representing the 38 classes of leaves. The optimizer is RMSprop with the learning rate α = 0.00005. The objective functions of the discriminator include Wasserstein loss function, gradient penalty function, and cross-entropy function as Eq. (3). We have conducted numerical experiments and analyses to tune the parameter *ε* in Eq. (3). The results showed that the quality of the synthetic images of WGAN-GP with LSR was better when *ε* was between 0.20 and 0.25. Therefore, the ε is set as 0.22 in this analysis.

**TABLE 2:** Architectures of the generator and the discriminator.

**Generator**		**Discriminator**	

**Type**	**Output Size**	**Type**	**Output Size**
Dense	8 × 8 × 128	Conv3-16(stride size = 2)	64 × 64 × 16
Up sampling	16 × 16 × 128	Conv3-32(stride size = 2)	32 × 32 × 32
Conv3-128	16 × 16 × 128	Zero padding	33 × 33 × 32
Up sampling	32 × 32 × 128	Conv3-64(stride size = 2)	17 × 17 × 64
Conv3-64	32 × 32 × 64	Conv3-128	17 × 17 × 128
Up sampling	64 × 64 × 64	Dense	1
Conv3-32	64 × 64 × 32	Dense	38
Up sampling	128 × 128 × 32		
Conv3-3	128 × 128 × 3		

*The convolutional layer parameters are denoted as “Conv(kernel size)- (number of channels).” Each convolutional layer is attached with a batch normalization layer and an activation layer (Leaky ReLU).*

As shown in Table 3, the CNN used to classify the images is the VGG16 with updated 128 × 128 × 3 input (Simonyan and Zisserman, 2014). The input layer is based on image RGB color space with a size of 128 × 128 × 3. The output layer has 38 neurons representing the 38 classes of leaves. The optimizer is RMSprop. The learning rate is 0.0001. The batch size is 100. All the above networks were built using the Keras framework (Chollet, 2015).

**TABLE 3:** Architecture of the CNN.

**Type**		**Output Size**	**Type**		**Output Size**
Block 1	Input Layer	128 × 128 × 3	Block 4	Conv3-512	16 × 16 × 512
	Conv3-64	128 × 128 × 64		Conv3-512	16 × 16 × 512
	Conv3-64	128 × 128 × 64		Conv3-512	16 × 16 × 512
	MaxPooling	64 × 64 × 64		MaxPooling	8 × 8 × 512
Block 2	Conv3-128	64 × 64 × 128	Block 5	Conv3-512	8 × 8 × 512
	Conv3-128	64 × 64 × 128		Conv3-512	8 × 8 × 512
	MaxPooling	32 × 32 × 128		Conv3-512	8 × 8 × 512
Block 3	Conv3-256	32 × 32 × 256		MaxPooling	4 × 4 × 512
	Conv3-256	32 × 32 × 256		AverPooling	1 × 1 × 512
	Conv3-256	32 × 32 × 256		Dense	512
	MaxPooling	16 × 16 × 256		Dense	38

### Experiment Design

To validate the proposed CNN framework, a comparative experiment using 90% of the original dataset (i.e., 39459 images) as train set and 10% (i.e., 4384 images) as the test set. The training accuracy achieved 99.9% while the testing accuracy achieved 99.8%. The results are comparable to the results obtained by Mohanty et al. (2016). It means that this framework can achieve a high prediction accuracy if there are enough data samples. Therefore, the proposed CNN framework can be used as the baseline model for this study. The influence of the CNN framework on the model performance can be ruled out.

Four numerical experiments have been designed, which used 873 training images and 4,384 testing images to keep consistency in the number of testing images. In Experiment I, the CNN is trained using the real dataset without any data augmentation. In Experiment II, the CNN is trained using real images with classic data augmentation methods. The classic augmentation methods include 360 rotation range, 0.3 width shift range, 0.3 height shift range, 0.3 zoom range, horizontal flip, and vertical flip. In Experiment III, the CNN is trained using the classic augmented data and the synthetic images generated by WGAN-GP without LSR. In each epoch, we use the trained generator to generate 30 new synthetic images for each category. In Experiment IV, the CNN is trained using the dataset generated by the proposed method. The training process is the same as that of the third experiment. It should be noted that, in Experiment III and IV, WGAN-GP is trained using the classic augmented data and then be used to generate synthetic images.

The number of images used for training in each epoch is shown in Table 4. In Experiment I, the 873 images used in each epoch are the same. In Experiment II, III and IV, the classic augmented images and synthetic images used in each epoch are new images that are generated randomly by the classical data augmentation methods and WGAN-GP, respectively. This paper implements the classic augmentation by using the ImageDataGenerator function from Keras package which replaces the original batch with the new, randomly transformed batch. Therefore, in Experiment II, III and IV, the number of original images used in each epoch is 0. The generator ran in parallel to the model for improved efficiency. For instance, this allows us to do real-time data augmentation on images on CPU in parallel to training our model on GPU.

**TABLE 4:** Number of images used for training in each epoch.

**Methods**	**# of original images**	**# of classic augmented images**	**# of synthetic images**
Experiment I	873	0	0
Experiment II	0	873	0
Experiment III	0	873	30*38
Experiment IV	0	873	30*38

To eliminate the influence of training time, the models are trained until the curve of training accuracy converges. This means the model performance cannot be improved by increasing the training time. Therefore, the number of epochs is set as 700. All experiments including the comparative experiment used the same testing dataset.

### Results and Comparisons

**FIGURE 5:**
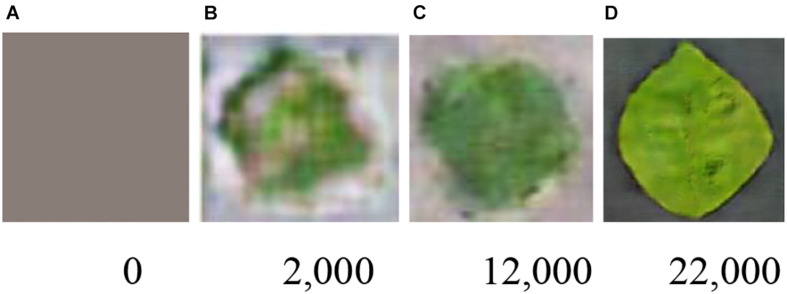
Synthetic images in different training stages of WGAN-GP-LSR (# of iterations) **(A)** 0, **(B)** 2,000, **(C)** 12,000, and **(D)** 22,000.

**FIGURE 6:**
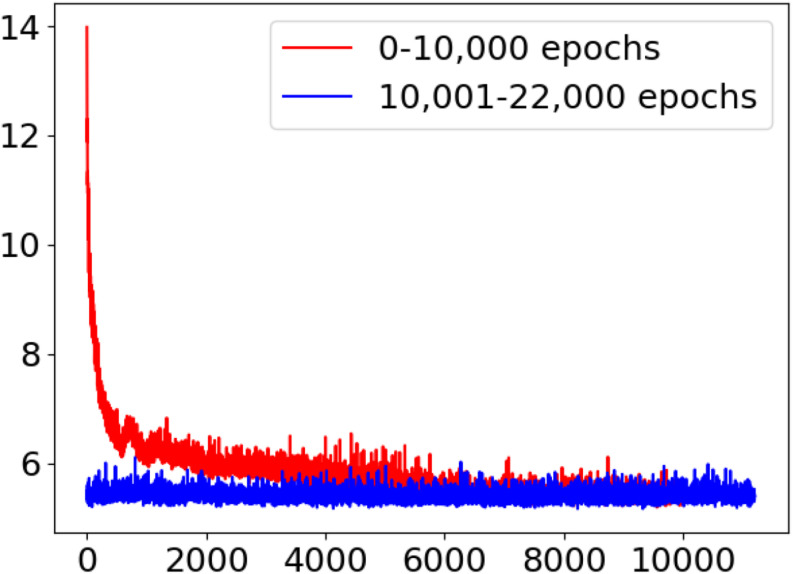
Train loss of WGAN-GP-LSR.

**FIGURE 7:**
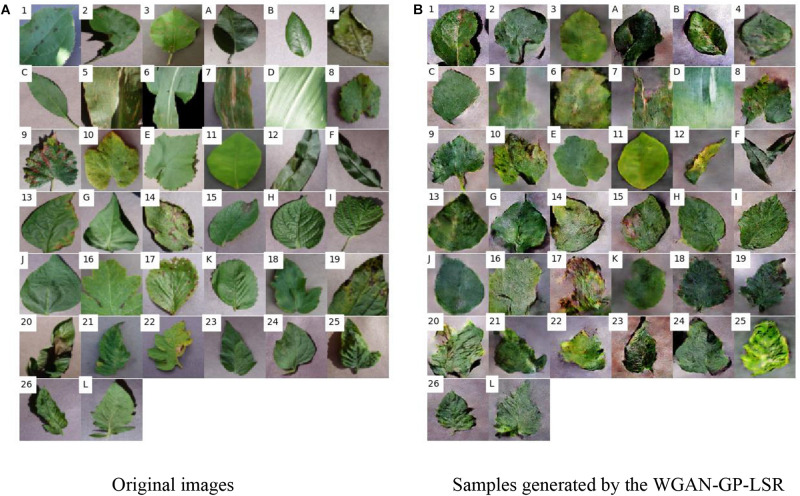
Original images and generated image samples. The images at the same location belong to the same class. The healthy classes are numbered from A to L. The diseased classes are numbered from 1–26. **(A)** Original images **(B)** Samples generated by the WGAN-GP-LSR.

The most important process is the training of the GAN. The training effectiveness of WGAN-GP-LSR can be illustrated by Figure 5. At the beginning, the output of the generator is just white noise. After 12,000 iterations, the outline of the leaf can be identified visually. At the 22,000th iteration, the shape of the leaf is much clearer. Figure 6 is the train loss curve of WGAN-GP-LSR. It can be seen that after 20,000, the Wasserstein distance, which is used to measure the distance between generated images and real images, converges. Figure 7A shows the real images drawn from 38 categories while Figure 7B shows the 38 samples generated by the regularized GAN. Each sample belongs to one unique class.

It can be found that the synthetic images look different from the original ones. There are two reasons for this. The first reason is that the synthetic images also contain information from other classes because of LSR. For example, for a classification problem of five classes, the ideal output of discriminator for a sample of class 1 should be [1,0, 0, 0, 0]. However, to increase the generalization ability of the model, the ideal output is expected to be [0.6, 0.1, 0.1, 0.1, 0.1]. This means the generated images also have small probabilities to be classified as other non-ground-truth classes. The second reason is that the WGAN-GP cannot generate perfect images that restore all details of real images due to the limited training set. The discriminator of WGAN only focuses on some specific regions (e.g., leaf shape, yellow spot, hole) that it can extract features from. Therefore, some information, such as background color and contrast degree, may be lost. However, the neural network can extract the right features to make predictions. The trained generator is used to generate additional images. Those images are mixed with real images and used as the input of the CNN.

**FIGURE 8:**
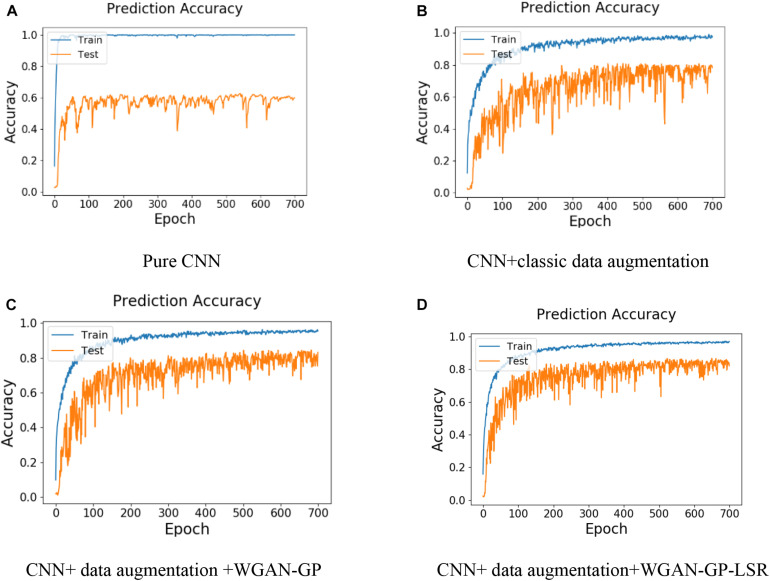
Results of the four numerical experiments **(A)** Pure CNN, **(B)** CNN+classic data augmentation, **(C)** CNN+ data augmentation +WGAN-GP, and **(D)** CNN+ data augmentation+WGAN-GP-LSR.

The results of the four experiments are shown in Figure 8. From Figure 8A, it can be found that after about 60 epochs, the training accuracy in Experiment I is close to 1 while the test accuracy is only about 60%. This is an indicator that the model is overfitted. It can be seen from Figure 8B that after using the classic data augmentation methods, the test accuracy in Experiment II is about 80%, which is 20% higher than that in Experiment I. Figure 8C shows the results of training CNN with classic data augmentation methods and synthetic data augmentation. After introducing the WGAN-GP, the test accuracy is improved by 1.9%. It proves that the synthetic images can increase the diversity of the dataset and improve the prediction accuracy. Since there are more training images, the curve of test accuracy is more stable than that in Experiment I and Experiment II. The results of Experiment IV is shown in Figure 8D. Compared to using WGAN-GP without LSR, the proposed method can improve the test accuracy by 2.1%, which validates the effectiveness of LSR.

Table 5 lists the training accuracy and test accuracy of the above four experiments. Compared to using CNN only, the proposed method improves the test accuracy by 21.6%. Compared to using CNN with classic data augmentation methods, the proposed method can improve the test accuracy by 4.2%. Compared to using CNN with classic data augmentation method and WGAN-GP, the proposed method can improve the test accuracy by 2.3%.

**TABLE 5:** Comparisons among four methods.

**Methods**	**Training accuracy**	**Test accuracy**
Pure CNN	100%	60.40%
CNN + classic data augmentation	90.08%	80.57%
CNN + classic data augmentation+ WGAN-GP	98.23%	82.41%
Proposed method (CNN + classic data augmentation+ WGAN-GP-LSR)	97.84%	84.78%

Table 6 includes the recall, precision, and F_1_ scores of 26 diseases. The top-5 F_1_ scores achieved by the proposed method are 0.91 on disease type 9 (Grape *Phaeomoniella* Spp.), 0.98 on disease type 11 (Orange *Candidatus Liberibacter*), 0.91 on disease type 14 (Potato *Alternaria solani*), 0.91 on disease type 16 (Squash *Erysiphe cichoracearum*) and 0.98 on disease type 25 (Tomato Mosaic Virus). Compared to using the CNN only, the advantages of the proposed method are dominant in terms of F_1_ score in almost all classes (i.e., 24 out of 26). For example, the proposed method improves F_1_ scores by 0.38 on disease type 8 (Grape *Guignardia bidwellii*), 0.57 on disease type 15 (Potato *Phytophthora infestans*) and 0.38 on disease type 21 (Tomato *Fulvia fulva*). The proposed method outperforms the CNN with classic data augmentation on most of the disease classes (i.e., 23 out of 26). Compared to using WGAN-GP without LSR, the proposed method performs much better on disease type 4 (Cherry *Podosphaera* Spp.) and disease type 14 (Potato Alternaria Solani). The average F_1_ score of the proposed method (i.e., 0.77) is higher than that of the CNN with classic data augmentation method (i.e., 0.71) and that of using WGAN-GP without LSR (i.e., 0.75).

**TABLE 6:** Recall, precision and F_1_ scores of 26 diseases (R: Recall; P: Precision; F: F_1_ score).

**Disease No.**	**Experiment I**			**Experiment II**			**Experiment III**			**Experiment IV**		
	**R**	**P**	**F**	**R**	**P**	**F**	**R**	**P**	**F**	**R**	**P**	**F**
**1**	0.17	0.89	0.29	0.48	0.54	0.51	0.33	0.88	0.48	0.46	0.60	0.52
2	0.59	0.61	0.60	0.86	0.77	0.81	0.66	0.84	0.74	0.93	0.72	0.81
3	0.37	0.41	0.39	0.50	0.88	0.64	0.70	0.75	0.72	0.30	0.56	0.39
4	0.41	0.85	0.55	0.88	0.43	0.58	0.43	0.93	0.59	0.71	0.96	0.82
5	0.50	0.42	0.46	0.69	0.56	0.62	0.47	0.68	0.56	0.56	0.72	0.63
6	0.53	0.80	0.64	0.89	0.90	0.89	0.96	0.73	0.83	0.92	0.86	0.89
7	0.87	0.65	0.75	0.78	0.76	0.77	0.61	0.91	0.73	0.81	0.79	0.80
8	0.28	0.40	0.33	0.94	0.45	0.61	0.69	0.87	0.77	0.74	0.69	0.71
9	0.92	0.55	0.69	0.67	0.85	0.75	0.91	0.91	0.91	0.91	0.91	0.91
10	0.51	0.84	0.63	0.62	0.95	0.75	0.58	0.91	0.71	0.60	0.98	0.74
11	0.96	0.66	0.78	0.93	0.98	0.95	0.96	0.95	0.96	0.99	0.97	0.98
12	0.66	0.93	0.77	0.95	0.64	0.77	0.90	0.90	0.90	0.95	0.80	0.87
13	0.73	0.43	0.54	0.85	0.55	0.67	0.81	0.71	0.76	0.93	0.53	0.68
14	0.52	0.75	0.61	0.84	0.65	0.74	0.35	0.93	0.50	0.89	0.94	0.91
15	0.12	0.78	0.21	0.79	0.54	0.64	0.72	0.95	0.82	0.69	0.89	0.78
16	0.57	0.98	0.72	0.94	0.88	0.91	0.94	0.82	0.88	0.86	0.97	0.91
17	0.88	0.78	0.83	0.71	0.63	0.67	0.86	0.84	0.85	0.89	0.75	0.82
18	0.28	0.62	0.38	0.62	0.86	0.72	0.84	0.79	0.81	0.66	0.98	0.79
19	0.22	0.65	0.32	0.53	0.52	0.52	0.76	0.57	0.65	0.62	0.66	0.64
20	0.43	0.59	0.50	0.62	0.73	0.67	0.67	0.77	0.72	0.70	0.75	0.73
21	0.29	0.54	0.37	0.54	0.78	0.64	0.81	0.92	0.86	0.81	0.70	0.75
22	0.65	0.57	0.61	0.52	0.89	0.66	0.94	0.52	0.67	0.76	0.70	0.73
23	0.54	0.67	0.60	0.63	0.82	0.71	0.58	0.97	0.73	0.72	0.92	0.81
24	0.60	0.45	0.52	0.31	0.73	0.44	0.93	0.53	0.67	0.81	0.67	0.73
25	0.95	0.74	0.83	0.89	0.98	0.94	0.97	0.89	0.92	0.99	0.97	0.98
26	0.88	0.62	0.73	0.92	1.00	0.96	0.85	0.92	0.88	0.92	0.80	0.86

When comparing the recall and the precision of each disease type, specific patterns of the models can be observed. For example, the difference between the recall and the precision of the disease type 10 (Grape *Pseudocercospora vitis*) is significantly different for all four models. The recall is 0.51∼0.6 while the precision is 0.84∼0.98. This means only a small number of images that have type 10 disease are classified as disease type 10. However, most of the images predicted that are classified to be type 10 are correctly labeled. The model might be confused between disease type 10 and other diseases, so it set a high standard for the classification of type 10. Therefore, the prediction of disease type 10 is highly reliable but the sensitivity of the model is low since the false negative predictions are high.

Since the objective of the training process is to improve the total prediction accuracy over all disease classes, it is not guaranteed that the proposed method will outperform other models in all categories. For example, the F_1_ score of disease type 3 (Apple *Gymnosporangium juniperi-virginianae*) in Experiment IV is much lower than that of other diseases. The reason is that the disease is more likely to be predicted as corn fungus diseases by the model. The comparison between the recall and the precision of each disease type can help to gain additional insights into the models and make the right decision according to different situations.

Table 7 lists the recall, precision and F_1_ scores of 12 healthy groups. The average F_1_ scores in the four experiments are 0.46, 0.76, 0.78 and 0.81, separately. However, all of the four models do not perform well for the classification of potato healthy leaves. Since there are only 15 testing images in this group, the reason might be that the distribution of the training set is not close to that of the testing set. Except for this, the F_1_ scores of most groups in Experiment II, III and IV are greater than 0.75.

**TABLE 7:** Recall, precision and F_1_ scores of 12 healthy groups (R: Recall; P: Precision; F: F_1_ score).

**Specie**	**Experiment I**			**Experiment II**			**Experiment III**			**Experiment IV**		
	**R**	**P**	**F**	**R**	**P**	**F**	**R**	**P**	**F**	**R**	**P**	**F**
Apple	0.37	0.72	0.49	0.93	1.00	0.96	0.81	0.92	0.86	0.89	0.94	0.91
Blueberry	0.44	0.74	0.55	0.91	1.00	0.95	0.76	0.97	0.85	0.84	0.8	0.82
Cherry	0.74	0.35	0.48	0.88	0.97	0.92	0.97	0.71	0.82	0.92	0.84	0.88
Corn	0.67	0.72	0.69	1.00	0.97	0.98	0.99	0.89	0.94	0.91	1.00	0.95
Grape	0.65	0.50	0.57	0.74	0.93	0.82	0.81	0.62	0.70	0.77	0.71	0.74
Peach	0.42	0.55	0.48	0.96	0.71	0.82	0.88	0.82	0.85	0.65	0.94	0.77
Pepper	0.73	0.17	0.28	0.79	0.83	0.81	0.86	0.68	0.76	0.94	0.8	0.86
Potato	0.13	0.22	0.16	0.13	0.67	0.22	0.40	1.00	0.57	0.53	0.47	0.50
Raspberry	0.37	0.76	0.50	0.90	0.68	0.77	0.27	1.00	0.43	0.8	0.85	0.82
Soybean	0.44	0.77	0.56	0.98	0.99	0.98	0.94	0.92	0.93	0.92	0.86	0.89
Strawberry	0.15	0.38	0.22	0.53	0.78	0.63	0.80	0.71	0.75	0.6	0.8	0.69
Tomato	0.54	0.70	0.61	0.85	0.92	0.88	0.86	0.97	0.91	0.86	0.97	0.91

## Conclusion

Plant disease recognition plays an important role in disease detection, mitigation, and management. Even though some deep learning methods have achieved good results in plant disease classification, the problem of the limited dataset is overlooked. In practice, it is time-consuming to collect and annotate data. The performance of CNN will drop dramatically if there is not enough training data. Therefore, a method for plant disease recognition under the limited training dataset is necessary.

In this paper, a CNN has been built for plant disease recognition, which can recognize multiple species and diseases. To address the overfitting problem caused by the limited training dataset, a GAN-based approach is proposed. The LSR method is also employed, which works by adding a regularization term to the loss function.

The experiments show that the proposed method can improve the prediction accuracy by 4.2% than the CNN with the classic data augmentation method. Compared with using the CNN only, the proposed method can improve the prediction accuracy by 24.4%. Compared with using the WGAN-GP without LSR, the proposed method can improve the prediction accuracy by 2.3%. Based on our work, plant disease classification can be conducted under the limited training dataset, which will bring benefits to the rapid diagnosis of plant diseases.

It should be noted that this proposed plant disease classification method is subject to a few limitations which suggest future research directions. First, significant computational resources are needed to train the GAN and generate new labeled images for training. This problem can be addressed using pre-trained models. Next, the proposed method still needs enough images to train the GAN. If the size of dataset is very small, it is not able to extract enough information to generate new labeled images. One potential solution to this is to introduce transfer learning techniques. Last, in this paper, we only used one CNN framework. In future, we will try different CNN frameworks and investigate the relationship between the size of the real image dataset and the effectiveness of the proposed method.

## Data Availability Statement

Publicly available datasets were analyzed in this study. This data can be found here: https://www.kaggle.com/emmarex/plantdisease, https://github.com/spMohanty/PlantVillage-Dataset/tree/master/raw/color.

## Author Contributions

LB worked on the data analysis, computational experiment, and drafting the manuscript. GH is the major professor for LB, she worked on the idea generation, refining the research approaches, and revising the manuscript. Both authors contributed to the article and approved the submitted version.

## Conflict of Interest

The authors declare that the research was conducted in the absence of any commercial or financial relationships that could be construed as a potential conflict of interest.

## Supplementary Material

Supplementary Materials The Python code of data processing and model training is available online at https://github.com/lbn-dev/WGAN_plant_diseases.

## References

[B1] Abu-Naser S. Kashkash K. Fayyad M. (2010). Developing an expert system for plant disease diagnosis. *J. Artif. Intell.* 3 269–276.

[B2] Arjovsky M. Chintala S. Bottou L. (2017). *Wasserstein Gan.* Available online at: https://arxiv.org/abs/1701.07875 (accessed January 26, 2017).

[B3] Barbedo J. G. A. (2018a). Factors influencing the use of deep learning for plant disease recognition. *Biosyst. Eng.* 172 84–91. 10.1016/j.biosystemseng.2018.05.013

[B4] Barbedo J. G. A. (2018b). Impact of dataset size and variety on the effectiveness of deep learning and transfer learning for plant disease classification. *Comput. Electron. Agric.* 153 46–53. 10.1016/j.compag.2018.08.013

[B5] Barbedo J. G. A. (2019). Plant disease identification from individual lesions and spots using deep learning. *Biosyst. Eng.* 180 96–107. 10.1016/j.biosystemseng.2019.02.002

[B6] Chollet F. (2015). *Keras.* Available online at: https://github.com/fchollet/keras (accessed August 18, 2020).

[B7] Dhakate M. Ingole A. (2015). “Diagnosis of pomegranate plant diseases using neural network,” in *Proceedings of the Fifth National Conference on Computer Vision, Pattern Recognition, Image Processing and Graphics (NCVPRIPG)*, (Patna: IEEE), 1–4.

[B8] Emeršic Ž. Štepec D. Štruc V. Peer P. (2017). Training convolutional neural networks with limited training data for ear recognition in the wild. *arXiv [Preprint]* 10.1109/FG.2017.123

[B9] Ferentinos K. P. (2018). Deep learning models for plant disease detection and diagnosis. *Comput. Electron. Agric.* 145 311–318. 10.1016/j.compag.2018.01.009

[B10] Ghazi M. M. Yanikoglu B. Aptoula E. (2017). Plant identification using deep neural networks via optimization of transfer learning parameters. *Neurocomputing* 235 228–235. 10.1016/j.neucom.2017.01.018

[B11] Goodfellow I. Pouget-Abadie J. Mirza M. Xu B. Warde-Farley D. Ozair S. (2014). “Generative adversarial nets,” in *Paper Presented at the Advances in Neural Information Processing Systems*, Montreal.

[B12] Grinblat G. L. Uzal L. C. Larese M. G. Granitto P. M. (2016). Deep learning for plant identification using vein morphological patterns. *Comput. Electron. Agric.* 127 418–424. 10.1016/j.compag.2016.07.003

[B13] Gu J. Wang Z. Kuen J. Ma L. Shahroudy A. Shuai B. (2018). Recent advances in convolutional neural networks. *Pattern Recognit.* 77 354–377.

[B14] Gulrajani I. Ahmed F. Arjovsky M. Dumoulin V. Courville A. C. (2017). “Improved training of wasserstein gans,” in *Paper Presented at the Advances in Neural Information Processing Systems*, (Cambridge, MA: MIT Press).

[B15] Guo J. Gould S. (2015). *Deep CNN Ensemble with Data Augmentation for Object Detection.* Available online at: https://arxiv.org/abs/1506.07224 (accessed June 24, 2015).

[B16] Hu G. Peng X. Yang Y. Hospedales T. M. Verbeek J. (2017). Frankenstein: learning deep face representations using small data. *IEEE Trans. Image Process.* 27 293–303. 10.1109/tip.2017.2756450 28952941

[B17] Hughes D. Salathé M. (2015). *An Open Access Repository of Images on Plant Health to Enable the Development of Mobile Disease Diagnostics.* Available online at: https://arxiv.org/abs/1511.08060 (accessed November 25, 2015).

[B18] Kamilaris A. Prenafeta-Boldú F. X. (2018). Deep learning in agriculture: a survey. *Comput. Electron. Agric.* 147 70–90. 10.1016/j.compag.2018.02.016

[B19] Lu Y. Yi S. Zeng N. Liu Y. Zhang Y. (2017). Identification of rice diseases using deep convolutional neural networks. *Neurocomputing* 267 378–384. 10.1016/j.neucom.2017.06.023

[B20] Ma J. Du K. Zheng F. Zhang L. Gong Z. Sun Z. (2018). A recognition method for cucumber diseases using leaf symptom images based on deep convolutional neural network. *Comput. Electron. Agric.* 154 18–24. 10.1016/j.compag.2018.08.048

[B21] Mirza M. Osindero S. (2014). *Conditional Generative Adversarial Nets.* Available online at: https://arxiv.org/abs/1411.1784 (accessed November 6, 2014).

[B22] Mohanty S. P. Hughes D. P. Salathé M. (2016). Using deep learning for image-based plant disease detection. *Front. Plant Sci.* 7:1419. 10.3389/fpls.2016.01419 27713752 PMC5032846

[B23] Naresh Y. Nagendraswamy H. (2016). Classification of medicinal plants: an approach using modified LBP with symbolic representation. *Neurocomputing* 173 1789–1797. 10.1016/j.neucom.2015.08.090

[B24] Nazki H. Yoon S. Fuentes A. Park D. S. (2020). Unsupervised image translation using adversarial networks for improved plant disease recognition. *Comput. Electron. Agric.* 168:105117 10.1016/j.compag.2019.105117

[B25] Papon J. Schoeler M. (2015). “Semantic pose using deep networks trained on synthetic RGB-D,” in *Proceedings of the IEEE International Conference on Computer Vision*, (Santiago: IEEE).

[B26] Patil J. K. Kumar R. (2011). Advances in image processing for detection of plant diseases. *J. Adv. Bioinform. Appl. Res.* 2 135–141.

[B27] Pereyra G. Tucker G. Chorowski J. Kaiser Ł Hinton G. (2017). *Regularizing Neural Networks by Penalizing Confident Output Distributions.* Available online at: https://arxiv.org/abs/1701.06548 (accessed January 23, 2017).

[B28] Radford A. Metz L. Chintala S. (2015). *Unsupervised Representation Learning with Deep Convolutional Generative Adversarial Networks.* Available online at: https://arxiv.org/abs/1511.06434 (accessed November 19, 2015).

[B29] Sankaran S. Mishra A. Ehsani R. Davis C. (2010). A review of advanced techniques for detecting plant diseases. *Comput. Electron. Agric.* 72 1–13. 10.1016/j.compag.2010.02.007

[B30] Simonyan K. Zisserman A. (2014). *Very Deep Convolutional Networks for Large-Scale Image Recognition.* Available online at: https://arxiv.org/abs/1409.1556 (accessed September 4, 2014).

[B31] Sladojevic S. Arsenovic M. Anderla A. Culibrk D. Stefanovic D. (2016). Deep neural networks based recognition of plant diseases by leaf image classification. *Comput. Intell. Neurosci.* 2016:3289801. 10.1155/2016/3289801 PMC4934169 27418923

[B32] Strange R. N. Scott P. R. (2005). Plant disease: a threat to global food security. *Annu. Rev. Phytopathol.* 43 83–116. 10.1146/annurev.phyto.43.113004.133839 16078878

[B33] Szegedy C. Vanhoucke V. Ioffe S. Shlens J. Wojna Z. (2016). “Rethinking the inception architecture for computer vision,” in *Proceedings of the IEEE Conference on Computer Vision and Pattern Recognition*, (Las Vegas, NV: IEEE).

[B34] Xie L. Wang J. Wei Z. Wang M. Tian Q. (2016). “Disturblabel: regularizing CNN on the loss layer,” in *Proceedings of the IEEE Conference on Computer Vision and Pattern Recognition*, (Las Vegas, NV: IEEE).

[B35] Zhang S. Wang Z. (2016). Cucumber disease recognition based on global-local singular value decomposition. *Neurocomputing* 205 341–348. 10.1016/j.neucom.2016.04.034

