# Supplementary material for: Improving Image-Based Plant Disease Classification With Generative Adversarial Network Under Limited Training Set
Source: Front Plant Sci. 2020 Dec 4;11:583438. doi: 10.3389/fpls.2020.583438 (PMC7746658; doi:10.3389/fpls.2020.583438)
Supplement: Supplementary Materials — The Python code of data processing and model training is available online at https://github.com/lbn-dev/WGAN_plant_diseases. [file Data_Sheet_1.pdf]

Appendix

Test confusion matrix of Experiment I

	1	2	3	4	5	6	7	8	9	10	11	12	13	14	15	16	17	18	19	20	21	22	23	24	25	26	A	B	C	D	E	F	G	H	I	J	K	L
1	8	3	0	0	0	0	0	0	1	0	7	1	4	0	0	0	0	3	0	0	7	1	0	1	0	0	0	2	0	2	0	0	6	0	0	0	0	0
2	0	34	0	0	0	0	0	1	0	0	2	0	3	0	0	0	0	0	0	0	0	1	0	0	0	0	0	0	1	0	0	0	16	0	0	0	0	0
3	0	0	11	0	3	0	2	1	1	1	2	2	0	0	0	0	0	7	0	0	0	0	0	0	0	0	0	0	0	0	0	0	0	0	0	0	0	0
4	0	0	0	40	0	0	0	9	1	1	12	0	3	0	0	2	0	0	0	0	0	0	2	0	6	0	1	1	1	0	0	0	9	0	0	5	0	5
5	0	0	0	1	16	0	7	0	5	0	1	0	0	0	0	0	0	1	0	1	0	0	0	0	0	0	0	0	0	0	0	0	0	0	0	0	0	0
6	0	0	2	0	13	48	13	0	6	0	1	0	0	0	0	0	0	0	0	2	0	0	0	0	0	0	0	0	0	0	0	0	1	0	0	4	0	0
7	0	0	0	0	4	0	60	0	1	0	1	0	0	0	0	0	1	0	0	1	0	0	0	0	0	0	0	0	1	0	0	0	0	0	0	0	0	0
8	0	1	0	0	0	0	0	26	43	0	2	0	7	0	0	0	0	4	1	0	0	4	0	0	4	0	0	0	0	0	0	0	2	0	0	0	0	0
9	0	1	0	0	0	0	0	1	108	0	0	0	1	0	0	0	2	0	1	1	0	0	0	1	1	0	0	0	0	0	0	0	0	0	0	0	0	0
10	0	0	0	0	0	0	0	1	1	46	2	0	6	4	0	0	3	2	0	0	0	0	2	0	9	0	0	0	0	0	0	0	2	0	0	6	0	6
11	0	0	0	0	0	1	0	0	0	6	449	2	2	0	0	0	0	0	0	0	0	6	0	0	0	0	0	1	0	0	0	0	0	0	0	0	0	0
12	0	5	1	0	0	0	0	0	2	1	19	124	5	1	1	0	0	0	4	0	0	0	0	0	0	0	1	0	0	0	0	3	20	0	0	0	0	0
13	0	1	0	0	0	1	0	1	8	0	0	0	70	0	0	0	0	0	0	0	0	3	0	3	1	0	0	0	2	0	0	0	6	0	0	0	0	0
14	0	0	1	1	1	0	2	0	0	0	0	0	4	42	0	0	8	0	0	14	0	6	0	0	0	0	0	0	0	0	0	0	0	0	0	0	0	2
15	1	0	0	0	0	0	1	6	0	0	4	0	0	0	7	0	1	3	0	14	2	3	4	7	1	0	0	0	0	0	0	0	4	0	0	0	0	0
16	0	0	0	3	1	0	0	3	0	0	4	0	0	0	0	96	0	2	0	0	0	0	1	2	18	0	0	0	0	6	0	0	13	0	0	7	6	6
17	0	0	0	0	0	0	0	0	1	0	0	0	2	0	0	0	57	0	0	1	0	3	0	0	0	0	0	0	1	0	0	0	0	0	0	0	0	0
18	0	0	0	1	0	0	0	0	3	0	55	3	4	0	0	0	0	45	1	0	0	3	0	0	30	0	2	7	0	0	0	0	2	7	0	0	0	0
19	0	0	0	0	0	2	0	2	6	0	4	1	4	1	0	0	0	2	20	3	1	6	1	9	22	0	0	0	0	0	0	0	9	0	0	0	0	0
20	0	0	12	1	0	8	1	0	1	0	8	0	4	5	1	0	0	3	0	61	3	13	1	4	11	0	0	0	2	0	0	0	3	0	0	0	0	0
21	0	0	0	0	0	0	0	0	0	0	2	0	5	3	0	0	0	0	3	1	20	12	1	1	13	0	3	0	0	0	0	0	6	0	0	0	0	0
22	0	1	0	0	0	0	0	0	3	0	1	1	16	0	0	0	1	0	1	2	2	89	0	3	0	1	0	0	3	0	0	0	12	0	0	0	0	0
23	0	0	0	0	0	0	0	0	0	0	8	0	8	0	0	0	0	0	0	0	1	0	80	10	6	2	0	0	0	0	0	0	18	0	0	2	2	12
24	0	0	0	0	0	0	0	0	0	0	3	0	0	0	0	0	0	0	0	0	1	4	14	73	1	5	0	0	1	0	0	0	13	0	0	6	0	0
25	0	0	0	0	0	0	0	0	0	0	14	0	3	0	0	0	0	0	0	0	0	0	1	0	396	0	0	0	1	0	0	0	1	0	0	0	0	0
26	0	0	0	0	0	0	0	0	0	0	0	0	0	0	0	0	0	0	0	0	0	1	0	0	1	23	0	0	0	0	0	0	1	0	0	0	0	0
A	0	0	0	0	0	0	0	0	0	0	39	0	0	0	0	0	0	1	0	3	0	0	1	1	1	0	60	3	1	10	0	5	26	0	0	11	0	0
B	0	9	0	0	0	0	0	0	3	0	0	0	1	0	0	0	0	0	0	0	0	0	0	1	0	0	2	51	0	0	6	1	41	0	0	0	2	0
C	0	0	0	0	0	0	0	0	0	0	11	0	0	0	0	0	0	0	0	0	0	0	0	0	0	0	0	0	56	0	0	0	9	0	0	0	0	0
D	0	1	0	0	0	0	6	3	3	0	6	0	0	0	0	0	0	0	0	0	0	0	0	0	5	0	0	0	0	57	0	0	4	0	0	0	0	0
E	0	0	0	0	0	0	0	0	0	0	0	0	0	0	0	0	0	0	0	0	0	0	0	0	0	0	0	0	5	0	20	0	6	0	0	0	0	0
F	0	0	0	0	0	0	0	0	0	0	5	0	0	0	0	0	0	0	0	0	0	0	0	0	0	0	0	0	0	0	0	11	9	0	0	1	0	0
G	0	0	0	0	0	0	0	0	0	0	4	0	4	0	0	0	0	0	0	0	0	0	0	2	6	0	0	0	5	0	0	0	77	0	0	7	0	0
H	0	0	0	0	0	0	0	0	0	0	0	0	0	0	0	0	0	0	0	0	0	0	0	0	0	3	0	0	0	0	0	0	8	2	1	1	0	0
I	0	0	0	0	0	0	0	10	0	0	0	0	0	0	0	0	0	0	0	0	0	0	0	0	2	0	0	0	6	0	0	0	14	0	19	0	0	0
J	0	0	0	0	0	0	0	0	0	0	8	0	0	0	0	0	0	0	0	0	0	0	0	21	0	0	11	0	67	0	14	0	91	0	0	166	0	0
K	0	0	0	0	0	0	0	1	0	0	0	0	0	0	0	0	0	0	0	0	0	0	0	0	0	0	0	4	3	0	0	0	22	0	4	0	6	0
L	0	0	0	0	0	0	0	0	0	0	8	0	5	0	0	0	0	0	0	0	0	0	11	23	0	3	3	0	4	4	0	0	1	0	1	0	0	73

Test confusion matrix of Experiment II

	1	2	3	4	5	6	7	8	9	10	11	12	13	14	15	16	17	18	19	20	21	22	23	24	25	26	A	B	C	D	E	F	G	H	I	J	K	L
1	22	1	0	0	0	0	0	2	1	0	0	10	0	4	1	2	0	0	0	0	3	0	0	0	0	0	0	0	0	0	0	0	0	0	0	0	0	0
2	0	50	0	2	0	0	0	2	0	0	0	2	1	0	0	0	0	0	0	0	0	0	0	0	0	0	0	0	0	0	0	1	0	0	0	0	0	0
3	1	2	15	1	3	2	1	0	0	0	0	2	3	0	0	0	0	0	0	0	0	0	0	0	0	0	0	0	0	0	0	0	0	0	0	0	0	0
4	0	0	0	86	0	0	0	2	0	0	0	2	3	0	0	0	0	0	0	1	0	0	0	0	0	0	0	0	0	0	0	0	0	0	4	0	0	0
5	0	0	0	1	22	2	6	0	0	0	0	0	1	0	0	0	0	0	0	0	0	0	0	0	0	0	0	0	0	0	0	0	0	0	0	0	0	0
6	1	0	0	0	0	80	4	0	0	0	0	0	5	0	0	0	0	0	0	0	0	0	0	0	0	0	0	0	0	0	0	0	0	0	0	0	0	0
7	0	0	0	0	10	4	54	0	0	0	0	0	1	0	0	0	0	0	0	0	0	0	0	0	0	0	0	0	0	0	0	0	0	0	0	0	0	0
8	0	0	0	0	0	0	1	88	3	0	0	2	0	0	0	0	0	0	0	0	0	0	0	0	0	0	0	0	0	0	0	0	0	0	0	0	0	0
9	0	2	0	0	1	0	4	27	78	0	0	0	2	1	0	0	0	0	2	0	0	0	0	0	0	0	0	0	0	0	0	0	0	0	0	0	0	0
10	0	0	0	4	0	0	0	9	0	56	1	0	0	0	2	4	11	0	1	1	0	0	0	0	0	0	0	0	0	0	0	1	0	0	0	0	0	0
11	0	0	0	12	0	0	0	4	0	0	434	5	3	0	0	0	0	3	0	0	2	0	0	0	1	0	0	0	1	0	0	1	1	0	0	0	0	0
12	0	0	0	3	0	0	0	4	0	0	1	178	0	0	0	0	0	0	0	0	0	0	0	0	0	0	0	0	0	0	0	1	0	0	0	0	0	0
13	1	1	0	0	0	0	0	4	0	0	0	4	82	0	0	0	1	1	2	0	0	0	0	0	0	0	0	0	0	0	0	0	0	0	0	0	0	0
14	1	0	0	1	1	1	0	0	0	0	0	1	0	68	2	2	2	0	0	0	1	1	0	0	0	0	0	0	0	0	0	0	0	0	0	0	0	0
15	0	0	0	4	0	0	0	0	0	0	0	0	0	4	46	0	0	0	1	3	0	0	0	0	0	0	0	0	0	0	0	0	0	0	0	0	0	0
16	0	0	0	5	0	0	0	0	0	0	0	0	0	0	0	158	0	1	0	2	0	0	0	0	0	0	0	0	0	0	0	0	0	0	0	0	0	2
17	0	0	0	0	0	0	0	0	7	0	0	1	1	6	2	2	46	0	0	0	0	0	0	0	0	0	0	0	0	0	0	0	0	0	0	0	0	0
18	0	0	0	12	2	0	0	14	0	0	1	13	2	5	0	0	0	101	8	1	0	1	0	0	1	0	0	0	0	0	0	1	0	0	0	1	0	0
19	2	2	0	7	0	0	0	11	2	0	0	3	1	1	3	0	0	1	49	5	0	1	0	5	0	0	0	0	0	0	0	0	0	0	0	0	0	0
20	0	0	2	3	0	0	0	2	0	0	0	19	5	4	9	2	0	2	2	88	1	0	0	0	0	0	0	0	0	0	0	0	3	0	0	0	0	0
21	1	0	0	3	0	0	0	5	0	0	0	6	0	0	2	0	0	1	4	1	38	6	1	1	1	0	0	0	0	0	0	0	0	0	0	0	0	0
22	2	2	0	0	0	0	1	3	0	3	0	7	11	6	0	0	13	1	10	1	2	71	0	2	0	0	0	0	0	0	1	0	0	0	0	0	0	0
23	0	0	0	2	0	0	0	3	0	0	1	4	1	0	9	5	0	0	6	4	0	0	94	1	5	0	0	0	0	3	0	0	0	0	5	0	4	2
24	0	0	0	11	0	0	0	9	0	0	0	7	4	4	5	0	0	1	2	11	0	0	18	38	0	0	0	0	0	0	0	3	1	0	0	1	0	6
25	1	0	0	2	0	0	0	5	0	0	5	1	10	0	0	1	0	4	7	1	2	0	2	1	370	0	0	0	1	0	0	0	2	0	1	0	0	0
26	0	0	0	0	0	0	0	0	0	0	0	1	0	0	0	0	0	0	0	0	0	0	0	1	0	24	0	0	0	0	0	0	0	0	0	0	0	0
A	3	0	0	1	0	0	0	1	0	0	0	1	0	0	0	1	0	1	0	2	0	0	0	0	0	0	151	0	0	0	0	0	0	0	0	1	0	0
B	6	2	0	3	0	0	0	0	0	0	0	0	0	0	0	0	0	0	0	0	0	0	0	0	0	0	0	106	0	0	0	0	0	0	0	0	0	0
C	0	1	0	0	0	0	0	0	0	0	0	0	0	0	0	0	0	0	0	0	0	0	0	0	0	0	0	0	67	0	0	2	6	0	0	0	0	0
D	0	0	0	0	0	0	0	0	0	0	0	0	0	0	0	0	0	0	0	0	0	0	0	0	0	0	0	0	0	85	0	0	0	0	0	0	0	0
E	0	1	0	5	0	0	0	0	0	0	0	2	0	0	0	0	0	0	0	0	0	0	0	0	0	0	0	0	0	0	23	0	0	0	0	0	0	0
F	0	0	0	0	0	0	0	0	0	0	0	1	0	0	0	0	0	0	0	0	0	0	0	0	0	0	0	0	0	0	0	25	0	0	0	0	0	0
G	0	0	0	2	0	0	0	1	0	0	0	4	7	0	0	0	0	0	0	0	0	0	0	3	0	0	0	0	0	0	0	0	83	0	2	1	2	0
H	0	0	0	3	0	0	0	0	0	0	0	0	2	0	1	0	0	0	0	0	0	0	0	0	0	0	0	0	0	0	0	0	3	2	4	0	0	0
I	0	0	0	3	0	0	0	0	0	0	0	1	0	0	0	1	0	0	0	0	0	0	0	0	0	0	0	0	0	0	0	0	0	0	46	0	0	0
J	0	1	0	2	0	0	0	0	1	0	0	0	1	0	0	1	0	0	0	0	0	0	0	0	0	0	0	0	0	0	1	0	0	0	1	370	0	0
K	0	0	0	6	0	0	0	0	0	0	0	1	3	0	1	1	0	0	0	0	0	0	0	0	0	0	0	0	0	0	0	0	1	1	5	0	21	0
L	0	0	0	16	0	0	0	0	0	0	0	0	0	2	2	0	0	0	0	0	0	0	0	0	0	0	0	0	0	0	0	0	0	0	0	0	0	116

Test confusion matrix of Experiment III

	1	2	3	4	5	6	7	8	9	10	11	12	13	14	15	16	17	18	19	20	21	22	23	24	25	26	A	B	C	D	E	F	G	H	I	J	K	L
1	15	3	0	0	0	0	0	1	0	0	0	8	2	0	0	0	0	0	5	0	1	7	0	2	0	0	0	0	0	0	1	0	0	0	0	1	0	0
2	1	38	0	0	0	0	0	2	0	0	0	0	2	0	0	0	0	0	0	0	0	4	0	0	0	0	0	0	1	0	7	0	0	0	0	3	0	0
3	0	0	21	0	1	1	0	0	0	0	0	0	1	0	0	0	0	1	0	0	0	5	0	0	0	0	0	0	0	0	0	0	0	0	0	0	0	0
4	0	0	0	42	0	0	0	0	0	2	6	0	1	0	2	24	0	1	0	0	0	0	0	2	10	0	1	1	2	3	0	0	1	0	0	0	0	0
5	0	0	0	0	15	10	2	0	0	0	0	0	1	0	0	1	0	1	0	1	0	1	0	0	0	0	0	0	0	0	0	0	0	0	0	0	0	0
6	0	0	0	0	0	86	1	0	0	0	0	0	1	0	0	0	0	0	0	0	0	2	0	0	0	0	0	0	0	0	0	0	0	0	0	0	0	0
7	0	0	4	0	6	12	42	0	0	0	0	0	0	0	0	1	0	0	0	3	0	0	0	0	0	0	0	0	0	1	0	0	0	0	0	0	0	0
8	0	0	0	0	0	0	0	65	9	1	0	6	0	0	0	1	0	2	4	0	0	6	0	0	0	0	0	0	0	0	0	0	0	0	0	0	0	0
9	0	0	0	0	0	0	1	2	106	0	0	1	2	1	0	0	0	0	3	0	0	1	0	0	0	0	0	0	0	0	0	0	0	0	0	0	0	0
10	0	0	0	0	0	0	0	3	0	52	0	0	2	0	0	1	9	5	7	0	0	8	0	0	2	0	0	0	0	0	1	0	0	0	0	0	0	0
11	0	0	0	0	0	0	0	0	0	0	450	1	3	0	0	1	0	2	0	0	0	0	0	0	4	0	0	0	1	3	1	0	0	0	0	0	1	0
12	0	0	2	0	0	0	0	1	0	0	2	168	5	0	0	0	0	4	1	0	1	2	0	0	0	0	0	0	0	0	1	0	0	0	0	0	0	0
13	0	0	0	0	0	0	0	0	0	0	1	0	78	0	0	0	0	0	0	0	0	15	0	1	0	0	0	0	0	0	1	0	0	0	0	0	0	0
14	0	0	0	0	0	3	0	0	0	1	0	0	2	28	0	0	0	0	2	9	0	36	0	0	0	0	0	0	0	0	0	0	0	0	0	0	0	0
15	0	0	0	1	0	0	0	0	0	0	1	0	0	0	42	1	0	1	2	5	0	2	0	2	0	0	0	0	0	0	0	0	1	0	0	0	0	0
16	0	0	1	1	0	0	0	0	0	0	0	0	0	0	0	158	0	2	0	0	0	0	0	1	1	0	0	0	0	1	0	0	0	0	0	0	1	2
17	0	0	0	0	0	0	0	0	1	0	0	0	1	0	0	0	56	0	3	0	0	4	0	0	0	0	0	0	0	0	0	0	0	0	0	0	0	0
18	0	0	0	0	0	0	0	0	0	0	1	0	0	0	0	1	0	137	6	2	0	3	0	1	10	0	0	0	0	0	0	0	0	0	0	2	0	0
19	0	0	0	0	0	0	0	1	0	1	0	1	1	0	0	0	0	3	71	1	0	6	0	5	3	0	0	0	0	0	0	0	0	0	0	0	0	0
20	0	0	0	0	0	6	0	0	0	0	1	0	4	1	0	4	0	7	7	95	0	2	0	5	7	0	0	0	0	0	0	0	3	0	0	0	0	0
21	0	0	0	0	0	0	0	0	0	0	0	0	0	0	0	0	0	0	1	2	57	5	1	2	2	0	0	0	0	0	0	0	0	0	0	0	0	0
22	0	0	0	0	0	0	0	0	0	0	0	0	1	0	0	0	0	1	0	2	1	128	0	2	0	0	0	0	1	0	0	0	0	0	0	0	0	0
23	0	0	0	0	0	0	0	0	0	0	3	0	2	0	0	0	0	0	4	1	1	0	87	42	3	1	0	0	1	0	0	0	1	0	0	1	0	2
24	0	0	0	0	0	0	0	0	0	0	0	0	1	0	0	0	0	2	3	0	0	3	0	112	0	0	0	0	0	0	0	0	0	0	0	0	0	0
25	0	0	0	0	0	0	0	0	0	0	6	0	0	0	0	0	0	0	5	0	0	1	0	2	402	0	0	0	0	0	0	0	0	0	0	0	0	0
26	0	0	0	0	0	0	0	0	0	0	0	0	0	0	0	0	0	0	0	0	1	2	0	1	0	22	0	0	0	0	0	0	0	0	0	0	0	0
A	1	0	0	1	0	0	0	0	0	0	0	0	0	0	0	0	0	5	0	2	0	0	0	3	0	0	131	1	0	1	0	4	0	0	0	13	0	0
B	0	4	0	0	0	0	0	0	0	0	0	0	0	0	0	0	0	0	0	0	0	1	0	1	1	0	10	89	0	0	0	1	4	0	0	0	6	0
C	0	0	0	0	0	0	0	0	0	0	1	0	0	0	0	0	0	0	0	0	0	0	0	0	0	0	0	0	74	0	0	0	0	0	0	1	0	0
D	0	0	0	0	0	0	0	0	0	0	0	0	0	0	0	0	0	0	0	0	0	0	0	0	0	0	0	0	0	84	0	0	0	0	0	1	0	0
E	0	0	0	0	0	0	0	0	0	0	0	0	0	0	0	0	0	0	0	0	0	0	0	0	0	0	0	0	4	0	25	0	0	0	0	2	0	0
F	0	0	0	0	0	0	0	0	0	0	0	2	0	0	0	0	0	0	0	0	0	0	0	0	0	0	0	0	1	0	0	23	0	0	0	0	0	0
G	0	0	0	0	0	0	0	0	0	0	0	0	0	0	0	0	0	0	0	0	0	0	0	6	1	0	0	0	1	0	1	0	90	0	0	5	1	0
H	0	0	0	0	0	0	0	0	0	0	0	0	0	0	0	0	0	0	1	0	0	0	0	3	0	0	0	0	0	0	0	0	3	6	0	1	1	0
I	0	0	0	0	0	0	0	0	0	0	0	0	0	0	0	0	2	0	0	0	0	0	0	0	8	1	0	0	1	0	0	0	20	0	14	2	3	0
J	0	0	0	0	0	0	0	0	0	0	0	0	0	0	0	0	0	0	0	0	0	0	1	1	0	0	0	0	16	0	2	0	4	0	0	354	0	0
K	0	0	0	0	0	0	0	0	0	0	0	0	0	0	0	0	0	0	0	0	0	0	0	0	0	0	0	1	1	1	0	0	5	0	0	0	32	0
L	0	0	0	0	0	0	0	0	0	0	0	0	0	0	0	0	0	0	0	0	0	0	1	18	0	0	0	0	0	0	0	0	0	0	0	0	0	117

Test confusion matrix of Experiment IV

	1	2	3	4	5	6	7	8	9	10	11	12	13	14	15	16	17	18	19	20	21	22	23	24	25	26	A	B	C	D	E	F	G	H	I	J	K	L
1	21	1	0	0	0	0	0	4	0	0	0	10	1	0	0	0	0	0	0	0	4	4	0	1	0	0	0	0	0	0	0	0	0	0	0	0	0	0
2	0	54	0	0	0	0	0	0	0	0	0	0	2	0	0	0	0	0	0	0	0	2	0	0	0	0	0	0	0	0	0	0	0	0	0	0	0	0
3	0	0	9	0	2	5	2	0	0	0	0	8	2	0	0	1	0	1	0	0	0	0	0	0	0	0	0	0	0	0	0	0	0	0	0	0	0	0
4	1	0	0	70	0	0	0	1	0	0	1	1	2	0	0	1	0	0	0	1	0	0	0	0	1	0	0	6	0	0	0	0	5	0	5	0	3	0
5	0	0	0	0	18	2	8	0	0	0	2	0	2	0	0	0	0	0	0	0	0	0	0	0	0	0	0	0	0	0	0	0	0	0	0	0	0	0
6	1	0	0	0	0	83	4	0	0	0	0	0	2	0	0	0	0	0	0	0	0	0	0	0	0	0	0	0	0	0	0	0	0	0	0	0	0	0
7	0	0	0	0	3	7	56	2	0	0	1	0	0	0	0	0	0	0	0	0	0	0	0	0	0	0	0	0	0	0	0	0	0	0	0	0	0	0
8	0	3	0	0	0	0	0	70	7	1	0	3	8	0	0	0	0	0	2	0	0	0	0	0	0	0	0	0	0	0	0	0	0	0	0	0	0	0
9	0	0	0	0	0	0	0	2	106	0	0	0	3	0	0	0	0	0	6	0	0	0	0	0	0	0	0	0	0	0	0	0	0	0	0	0	0	0
10	0	0	0	0	0	0	0	3	0	54	3	0	6	1	0	0	13	0	4	0	3	1	0	1	0	0	0	0	0	0	0	0	1	0	0	0	0	0
11	0	0	0	0	0	0	0	0	0	0	462	0	0	0	0	0	0	0	0	0	0	0	0	0	1	0	1	0	2	0	0	0	1	0	0	0	0	0
12	0	1	0	0	0	0	0	3	0	0	0	178	4	0	0	0	0	0	0	0	0	0	0	0	0	0	0	0	0	0	0	0	1	0	0	0	0	0
13	0	1	0	0	0	0	0	0	0	0	0	0	89	0	0	0	0	0	0	1	0	2	0	1	0	0	0	2	0	0	0	0	0	0	0	0	0	0
14	1	2	0	0	0	0	0	0	0	0	0	0	0	72	1	0	3	0	1	1	0	0	0	0	0	0	0	0	0	0	0	0	0	0	0	0	0	0
15	0	0	0	1	0	0	1	1	0	0	0	1	2	1	40	0	0	0	0	9	0	1	0	0	0	0	0	0	0	0	0	0	0	0	0	1	0	0
16	1	1	3	1	0	0	0	0	0	0	0	2	0	1	1	144	0	0	0	0	0	0	0	3	0	0	0	4	0	0	2	0	1	0	0	0	2	2
17	0	0	0	0	0	0	0	0	2	0	0	1	4	0	0	0	58	0	0	0	0	0	0	0	0	0	0	0	0	0	0	0	0	0	0	0	0	0
18	0	0	0	0	2	0	0	9	0	0	2	2	5	0	1	0	0	108	10	3	2	6	0	0	4	0	2	0	0	0	0	0	0	0	0	7	0	0
19	0	0	0	0	0	0	0	5	1	0	0	0	4	0	0	0	3	0	58	2	4	5	0	8	2	0	0	0	0	0	0	0	1	0	0	0	0	0
20	2	0	4	0	0	0	0	0	0	0	1	1	4	1	1	1	0	0	2	99	5	1	1	1	1	0	0	0	1	0	0	0	6	0	0	8	0	2
21	0	0	0	0	0	0	0	0	0	0	0	1	0	0	0	0	0	0	0	1	57	4	2	0	0	0	4	0	0	0	0	0	1	0	0	0	0	0
22	0	0	0	0	0	0	0	0	0	0	1	0	24	1	0	0	0	0	0	4	1	103	0	0	0	0	0	0	0	0	0	0	2	0	0	0	0	0
23	0	0	0	0	0	0	0	0	0	0	2	1	0	0	0	0	0	0	2	3	4	3	107	20	2	3	0	1	1	0	0	0	0	0	0	0	0	0
24	0	0	0	0	0	0	0	0	0	0	0	1	0	0	0	0	0	0	2	3	0	8	4	98	0	1	0	0	0	0	0	0	0	0	0	4	0	0
25	0	0	0	0	0	0	0	0	0	0	1	0	0	0	0	0	0	1	0	0	0	0	1	0	412	0	0	0	0	0	0	0	1	0	0	0	0	0
26	0	0	0	0	0	0	0	0	0	0	0	0	0	0	0	0	0	0	0	0	1	1	0	0	0	24	0	0	0	0	0	0	0	0	0	0	0	0
A	1	3	0	0	0	0	0	1	0	0	0	2	0	0	0	0	0	0	0	1	0	2	1	2	0	0	144	3	0	0	0	1	0	0	0	1	0	0
B	7	5	0	0	0	0	0	0	0	0	0	0	2	0	0	0	0	0	0	0	0	0	0	0	0	0	1	98	0	0	0	0	4	0	0	0	0	0
C	0	0	0	0	0	0	0	0	0	0	0	0	0	0	0	0	0	0	0	0	0	2	0	0	0	0	0	0	70	0	0	0	0	0	0	4	0	0
D	0	0	0	0	0	0	0	0	0	0	0	0	0	0	0	1	0	0	0	0	0	0	0	0	0	0	0	1	1	77	0	0	0	0	1	3	1	0
E	0	1	0	0	0	0	0	0	0	0	0	0	0	0	0	0	0	0	0	0	0	0	0	0	0	0	0	0	3	0	24	0	0	0	0	3	0	0
F	0	0	0	0	0	0	0	0	0	0	0	8	0	0	0	0	0	0	0	0	0	1	0	0	0	0	0	0	0	0	0	17	0	0	0	0	0	0
G	0	0	0	0	0	0	0	0	0	0	0	0	0	0	0	0	0	0	0	0	0	0	0	1	0	0	0	0	0	0	0	0	99	0	0	5	0	0
H	0	0	0	0	0	0	0	0	0	0	0	0	0	0	0	0	0	0	0	0	0	0	0	0	0	1	0	0	0	0	0	0	0	8	0	6	0	0
I	0	0	0	0	0	0	0	0	0	0	0	0	0	0	0	0	0	0	0	0	0	0	0	0	1	0	0	0	2	0	0	0	1	2	41	4	0	0
J	0	3	0	0	0	0	0	0	0	0	0	0	0	0	0	0	0	0	0	0	0	1	0	2	1	1	1	2	3	0	8	0	0	7	1	348	0	0
K	0	0	0	0	0	0	0	0	0	0	0	0	2	0	0	0	0	0	1	0	0	0	0	0	0	0	0	5	0	0	0	0	0	0	0	8	24	0
L	0	0	0	1	0	0	0	0	0	0	0	2	0	0	1	0	0	0	0	4	0	0	0	8	0	0	0	0	0	0	0	0	0	0	0	3	0	117
